# Tick Fauna and Associated *Rickettsia*, *Theileria*, and *Babesia* spp. in Domestic Animals in Sudan (North Kordofan and Kassala States)

**DOI:** 10.3390/microorganisms8121969

**Published:** 2020-12-11

**Authors:** Andrea Springer, Yassir Adam Shuaib, Makarim Habib Isaa, Malaz Isam-Eldin Ezz-Eldin, Abdinasir Yusuf Osman, Idris Ahmed Yagoub, Mohamed Abdalsalam Abdalla, Amel Omer Bakiet, Saad El-Tiab Mohmed-Noor, Sabine Schaper, Ramona Rieß, Gerhard Dobler, Christina Strube, Deon K. Bakkes, Lidia Chitimia-Dobler

**Affiliations:** 1 Institute for Parasitology, Centre for Infection Medicine, University of Veterinary Medicine Hannover, Buenteweg 17, 30559 Hanover, Germany; christina.strube@tiho-hannover.de; 2 College of Veterinary Medicine, Sudan University of Science and Technology, Hilat Kuku, Khartoum North 13321, Sudan; vet.aboamar@gmail.com (Y.A.S.); salamaa0002@gmail.com (M.A.A.); amel33@gmail.com (A.O.B.); 3 Veterinary Research Institute, Soba, Khartoum 11121, Sudan; makarim.habeeb@gmail.com (M.H.I.); yagoubidris@yahoo.com (I.A.Y.); 4 Ministry of Animal Resources, Kassala 22271, Kassala State, Sudan; Mezo8811@gmail.com; 5 The Royal Veterinary College, University of London, Hawkshead Lane, North Mymms, Hatfield AL9 7TA, Hertfordshire, UK; aosman@rvc.ac.uk; 6 Veterinary Ambulance, Khartoum 11111, Sudan; mongash2@gmail.com; 7 Bundeswehr Institute of Microbiology, 80937 Munich, Germany; SabineSchaper@bundeswehr.org (S.S.); ramonariess@bundeswehr.org (R.R.); gerharddobler@bundeswehr.org (G.D.); lydiachitimia@gmail.com (L.C.-D.); 8 Department of Parasitology, University of Hohenheim, 70599 Stuttgart, Germany; 9 Gertrud Theiler Tick Museum, Onderstepoort Veterinary Research, Pretoria 0001, South Africa; BakkesD@arc.agric.za; 10 Evolutionary Genomics Group, Department of Botany and Zoology, Stellenbosch University, Matieland, 7602 Stellenbosch, South Africa

**Keywords:** *Hyalomma*, *Amblyomma*, *Rhipicephalus*, *Rickettsia* spp., piroplasms, tick-borne diseases, vector-borne diseases

## Abstract

Ticks and tick-borne diseases (TBDs) have a major economic impact on animal production worldwide. In the present study, 2410 ticks were collected from January to August 2017 from livestock and other domestic animals in North Kordofan and Kassala, Sudan, for species identification and investigation of *Rickettsia* spp. and piroplasms, either individually or as pools containing up to 10 ticks by molecular methods. In total, 13 tick species were identified by morphology and 16S rDNA sequencing. The most frequent tick species were *Hyalomma impeltatum* (24.90%), *Rhipicephalus evertsi evertsi* (18.84%), *Amblyomma lepidum* (16.06%), and *Rhipicephalus camicasi* (12.49%). A pan-*Rickettsia* real-time PCR revealed an overall minimum infection rate (MIR) with *Rickettsia* spp. of 5.64% (136 positive tick pools/2410 total ticks). *Rickettsia africae* and *Rickettsia aeschlimannii* were the most frequently identified species by sequencing. Furthermore, the following highly pathogenic livestock parasites were detected: *Theileria annulata*, *Theileria lestoquardi*, *Theileria equi*, and *Babesia caballi*. The present study documented *Rhipicephalus afranicus* as well as *Rickettsia conorii israelensis*, *Rickettsia massiliae*, and *Babesia pecorum* for the first time in Sudan. These findings are significant for the animal production sector as well as in terms of One Health, as the detected *Rickettsia* spp. can cause serious illness in humans.

## 1. Introduction

Ticks are medically important ectoparasites of mammals, birds, reptiles, and amphibians. Their haematophagous feeding habit may result in blood loss and mechanical damage to the skin [1,2]. Furthermore, ticks can transmit many pathogens to humans and animals, including viruses (e.g., Crimean–Congo haemorrhagic fever (CCHF) virus), bacteria (e.g., *Rickettsia* spp.) protozoans (e.g., *Babesia* spp. and *Theileria* spp.) and helminths [3]. Ticks and tick-borne diseases (TBDs) result in economic losses that are estimated at USD 22–30 billion globally every year [4].

The burden and dynamics of ticks and TBDs are changing with the concurrent global climate change [5]. Moreover, anthropogenic factors can influence the biodiversity and population dynamics of ticks [5]. Consequently, a substantial increase in TBDs has been noted in certain regions, e.g., human cases of TBDs more than doubled in the United States from 2004 to 2016 [6]. Furthermore, some ticks have expanded their habitats to new exotic areas [7]. The introduction of novel tick species may lead to cascading effects on the local tick fauna and TBDs. This is clearly illustrated by the introduction of *Rhipicephalus microplus*, vector of the cattle parasites *Babesia bovis* and *Babesia bigemina*, into several African countries, which led to displacement of native *Rhipicephalus* spp. and may result in increased *Babesia* transmission [8,9].

Overall, the reported tick species in Africa belong to 10 different genera, including *Amblyomma*, *Hyalomma*, *Rhipicephalus*, *Ixodes*, *Argas*, and *Ornithodoros*, for example [10]. Tick-borne pathogens of livestock, such as the causative agents of theileriosis, babesiosis and heartwater, have been circulating on the continent, often with a considerable economic cost [11,12,13]. Additionally, zoonotic TBDs, including rickettsioses, pose a serious public health problem [14,15]. Therefore, a contemporary tick and TBD surveillance is relevant in African countries.

Sudan has a huge wealth of livestock that is estimated to be more than 100 million animals [16]. A substantial proportion of the people of Sudan own livestock for subsistence [17]. These people are vulnerable to the negative impact of animal diseases, including TBDs, and ticks. Previous studies have shown a high prevalence of *Theileria* spp. in Sudanese livestock, especially regarding the highly pathogenic *Theileria parva* in cattle [18] and *Theileria equi* in horses [19]. Furthermore, tick-transmitted *Rickettsia* spp., especially *Rickettisa africae*, which causes African tick-borne fever, have a high prevalence in the region and represent a public health hazard [15,20,21]. Moreover, in the past few years, changes in the tick fauna have been noted in Sudan [17], probably due to the continuous movement of animals in search of pastures and water as well as due to natural and anthropogenic changes in environment [17]. This highlights the need to monitor how the natural tick population is changing in Sudan, particularly in animal production areas. In this study, we collected 2410 ticks from livestock and other domestic animals in North Kordofan and Kassala states, Sudan, for species identification and investigation of *Rickettsia* spp., *Theileria* spp., and *Babesia* species.

## 2. Materials and Methods

### 2.1. Study Area

**Figure 1:**
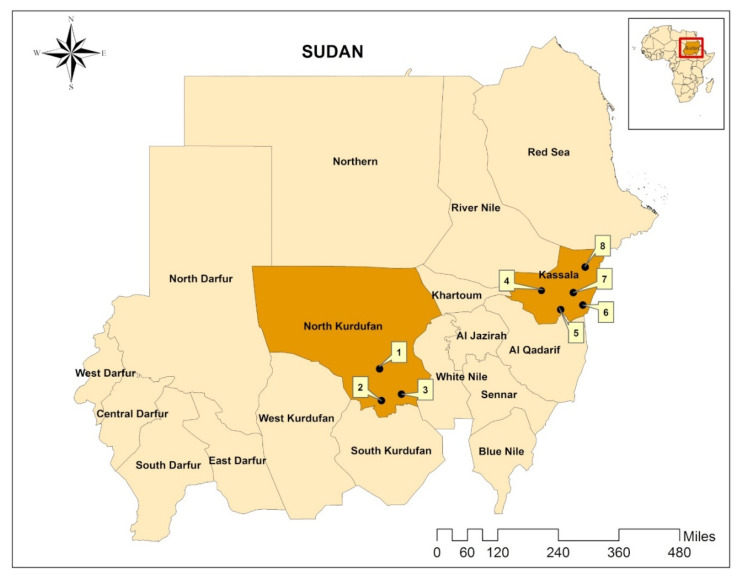
Map showing the location of Sudan in Africa (small map) and the location of the study areas in Sudan (North Kordofan and Kassala states, indicated by orange). Sampling was conducted in Sheikan (1), Al-Rahad (2), Um-Ruwabah (3), West Kassala (4), Kashm el Griba (5), Kassala (6), Aroomah (7) and Wagar (8). The map was created using ArcGIS v. 10 (esri Inc., Redlands, CA, USA).

North Kordofan state is located in the central part of Sudan between latitudes 11° and 16° N and longitudes 27° and 32° E, with a total area of 185,302 km^2^, while Kassala state is located in the eastern part of the country between latitude 14° and 17° N and longitude 34° and 37° E, covering an area of 42,282 km^2^ (Figure 1). Annual rainfall (up to 700 mm/year) is concentrated in a single relatively short season from June to September (hot rainy season), followed by a cold dry season from October to January and a hot dry season from February to May. Up to 40% of the states’ total land is cultivable and agriculture and livestock comprises up to 70% of the economic activities. The states have abundant fodder, grazing areas, and water sources, like seasonal rivers (e.g., Kour Abu-Habil in North Kordofan and Atbara and Gaash in Kassala) and hafeers (i.e., rainwater harvesting sites), during the rainy season. This supports a considerably large livestock population, which is estimated at 13,061,246 head in North Kordofan and 4,479,050 in Kassala [16]. Animals are raised in a mixture of farming systems, such as mixed crop–livestock, nomadic, sedentary, and semi-sedentary, for domestic consumption and for export to international markets (e.g., Gulf countries).

### 2.2. Tick Collection and Identification

In this cross-sectional study, tick specimens were collected from livestock and other domestic animals in the period from January to August 2017, in North Kordofan and Kassala states, Sudan [14]. Samples were collected from three localities in the state of North Kordofan, including Sheikan, Al-Rahad, and Um-Ruwabah, and from five localities in the state of Kassala, namely Aroomah, Wagar, Kassala, West Kassala, and Khashm el Griba (Figure 1). These localities were selected randomly and/or conveniently. In both areas, mostly sheep and goats were examined, followed by cattle, camels and dogs. Sampled animals were thoroughly examined for attached ticks by searching the head and ears, the neck (dewlap), the thoracic area and the abdomen, the udder or scrotum, the fore- and hindlimbs, perineum, and the tail. Attached ticks were either collected by hand- or forceps-picking and stored in 70% ethanol. All ticks collected from the same animal host were put into one tube. Tubes were labelled (location, animal species and date of collection) and sent to the Bundeswehr Institute of Microbiology, Munich, Germany, where ticks were identified to species level using morphological characteristics described by Apanaskevich and Horak [22], Apanaskevich and Horak [23], Apanaskevich and Horak [24], Apanaskevich, et al. [25], Voltzit and Keirans [26] and Walker, et al. [10,27].

### 2.3. Nucleic Acid Extraction

Total nucleic acid was extracted using the MagNA Pure LC RNA/DNA Kit (Roche, Mannheim, Germany) in a MagNA Pure LC instrument (Roche) according to the manufacturer’s instructions. Total nucleic acid was extracted from individual ticks or pools containing 2–10 specimens per pool, if the ticks shared the same developmental stage and species and were collected from the same animal. The extracted total nucleic acid was stored at −80 °C until use.

### 2.4. Molecular Tick Species Identification

Identification of ticks that were either damaged or fully engorged, and thus not reliably identifiable based on morphological criteria (*n* = 23), as well as confirmation of primary morphological determinations (*n* = 13), was achieved by 16S rDNA sequencing (250 bp fragment) and phylogenetic analysis. The gene was amplified using polymerase chain reaction (PCR) protocols and sequenced in both directions as previously described by Mangold, et al. [28]. Tick sequences generated in this study are available in GenBank (MT535883-MT535906). Additional sequences from GenBank were chosen to cover the range of *Rhipicephalus* and *Hyalomma* species that occur in Sudan and closely related species. As the prevalence of misidentified tick species among sequence data in GenBank is a growing problem, the selected sequences were derived from recent studies that included large-scale taxonomic investigations to verify identification by phylogenetic analysis and correlated morphology [29,30,31,32,33,34]. Sequence data were aligned using MAFFT (Q-INS-I, 200PAM/k = 2, Gap opening penalty 1.53) [35], and the final alignment comprised 265 nucleotide characters. The alignment was inspected manually to ensure sequences were in reading frame. Phylogenetic analysis was based on maximum likelihood with 1000 bootstrap replicates in MEGA v7.0.14 [36] using a TPM2u + F + G4 model determined by Bayesian Information Criterion calculations in W-IQ-TREE [37].

### 2.5. PCR for Rickettsia spp. and Piroplasms

For detection of *Rickettsia* spp., a pan-*Rickettsia* real-time PCR was used [38,39]. Positive samples were further subjected to *Rickettsia* species identification by amplification, sequencing in both directions and phylogenetic analysis of the 23S-5S intergenic spacer region (330 bp fragment) as described by Chitimia-Dobler, et al. [40]. Additional sequences from GenBank were chosen to cover the range of *Rickettsia* species that occur in Africa and Eurasia [41,42]. Sequence data were aligned using MAFFT (Q-INS-I, 200PAM/k = 2, Gap opening penalty 1.53) [35], and the final alignment comprised 403 nucleotide characters. The alignment was inspected manually to ensure sequences were in reading frame. Phylogenetic analysis was based on maximum likelihood with 1000 bootstrap replicates in MEGA v7.0.14 [36] using an HKY + F + G4 model determined by Bayesian Information Criterion calculations in W-IQ-TREE [37].

To identify whether the collected ticks were infected with piroplasms, the pools were tested for *Theileria* spp. and *Babesia* spp. DNA by amplifying a part of the 18S rDNA in a conventional PCR, using the primers BJ1 and BN2 [43], as described by Springer, et al. [44]. Obtained 18S rDNA amplicons were custom Sanger-sequenced (Microsynth Seqlab Sequencing Laboratories, Göttingen, Germany), or—in case of weak bands—ligated into the pCR™4-TOPO^®^ TA vector and cloned into One Shot Top10 chemically competent *Escherichia coli* (TOPO^®^ TA Cloning kit, Thermo Fisher Scientific GmbH, Dreieich, Germany). After plasmid extraction and purification (NucleoSpin Plasmid kit, Macherey-Nagel GmbH & Co. KG, Düren, Germany), the insert was custom Sanger-sequenced, as indicated above. *Rickettsia* spp. and piroplasms’ sequences generated in this study are available in GenBank under the accession numbers MW152276–MW152327 for *Rickettsia* spp., and MW131349–MW131365 for piroplasms. Minimum infection rates (MIRs) were calculated under the assumption of only one positive tick per pool (MIR = number of positive pools/total number of ticks).

## 3. Results

### 3.1. Identified Tick Species

In total, 2410 ticks, including 1301 from North Kordofan and 1109 from Kassala, were collected from cattle, sheep, goats, camels and horses. Based on morphological characteristics and 16S rDNA sequencing, 13 different tick species belonging to three genera were identified (i.e., *Hyalomma* (998/2410, 41.41%), *Amblyomma* (445/2410, 18.46%), and *Rhipicephalus* (967/2410, 40.12%)). Overall, *Hyalomma impeltatum* was most frequently identified (600/2410, 24.90%), followed by *Rhipicephalus evertsi evertsi* (454/2410, 18.84%), *Amblyomma lepidum* (387/2410, 16.06%), *Rhipicephalus camicasi* (301/2410, 12.49%), and *Hyalomma anatolicum* (185/2410, 7.68%). The remaining eight species were found in proportions ranging from 0.04% to 7.68%. Detailed data on tick species and the number of male, female and nymphal ticks of each species are presented in Table 1.

**Figure 2:**
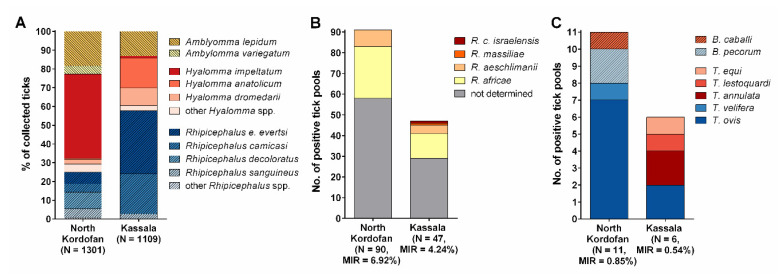
Species composition of ticks and tick-borne pathogens detected in North Kordofan and Kassala, Sudan. (**A**) Species composition of ticks collected from livestock and other domestic animals from January to August 2017. (**B**) *Rickettsia* species composition among pan-*Rickettsia* real-time PCR-positive tick pools. (**C**) Piroplasm species composition among PCR-positive tick pools. Pathogenic species are indicated in shades of red, while apathogenic species are shown in shades of blue. MIR = minimum infection rate.

In North Kordofan, *H. impeltatum* was most abundant (587/1301, 45.11%), followed by *A. lepidum* (241/1301, 18.52%) and *Rh. decoloratus* (116/1301, 8.92%) (Figure 2A). In contrast, *Rh. evertsi evertsi* (373/1109, 33.63%), *Rh. camicasi* (234/1109, 21.10%) and *H. anatolicum* (176/1109, 15.87%) were the main identified tick species in Kassala. *A. variegatum* (58/1301, 4.46%) and *H. truncatum* (6/1301, 0.46%) were found only in North Kordofan, while *Rh. afranicus* was found only in Kassala (1/1109, 0.09%).

**Figure 3:**
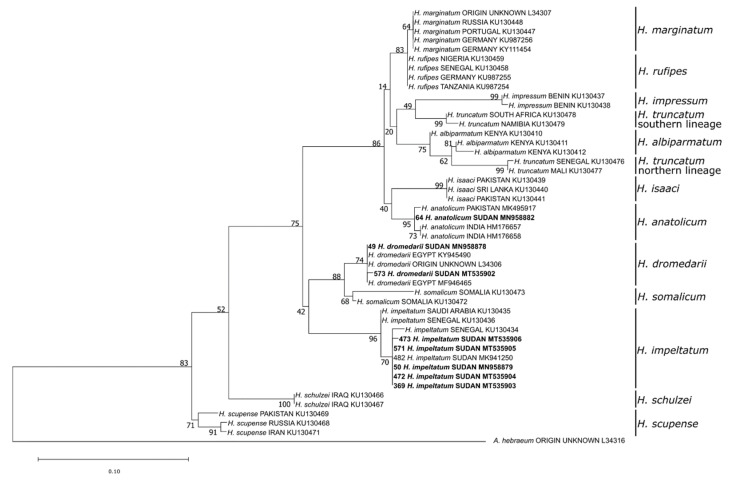
Maximum likelihood phylogenetic analysis of 16S rDNA sequences of *Hyalomma* spp. ticks using *Amblyomma hebraeum* as an outgroup. Bolded tip labels refer to sequences generated in this study, and sample ID, species/lineage names, country of origin and GenBank accession numbers are indicated. Nodal values indicate bootstrap support using 1000 replicates.

**Figure 4:**
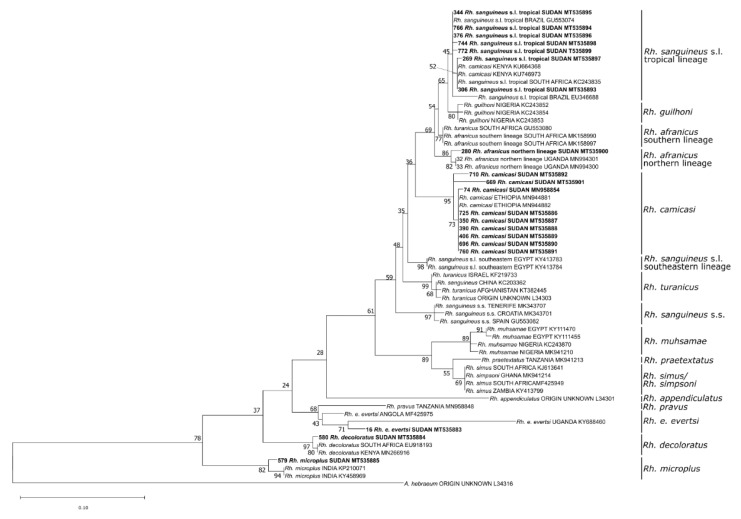
Maximum likelihood phylogenetic analysis of 16S rDNA sequences of *Rhipicephalus* spp. ticks using *Amblyomma hebraeum* as an outgroup. Bolded tip labels refer to sequences generated in this study, and sample ID, species/lineage names, country of origin, and GenBank accession numbers are indicated. Nodal values indicate bootstrap support using 1000 replicates.

Phylogenetic analysis of the 16S rDNA sequences confirmed the morphology-based species determination for eight damaged or fully engorged *Hyalomma* ticks, namely *H. anatolicum* (*n* = 1), *H. dromedarii* (*n* = 2), and *H. impeltatum* (*n* = 5) (Figure 3). Moreover, the species of 18 *Rhipicephalus* ticks, including *Rh. sanguineus* s.l. tropical lineage (*n* = 7), *Rh. afranicus* (*n* = 1), *Rh. camicasi* (*n* = 7), *Rh. evertsi evertsi* (*n* = 1), *Rh. decoloratus* (*n* = 1) and *Rh. microplus* (*n* = 1), could only be identified by Sanger-sequencing (Figure 4).

### 3.2. Prevalence of Tick-Borne Pathogens

#### 3.2.1. *Rickettsia* Species

In total, 783 tick pools were tested for *Rickettsia* species by real-time PCR. Of these, 136 were *Rickettsia*-positive, resulting in an MIR of 5.64% (136/2410). *Rickettsia* DNA was detected in 11 out of 13 tick species (Table 1). *Rickettsia* species composition among the positive tick pools from North Kordofan and Kassala is shown in Figure 2B.

**Figure 5:**
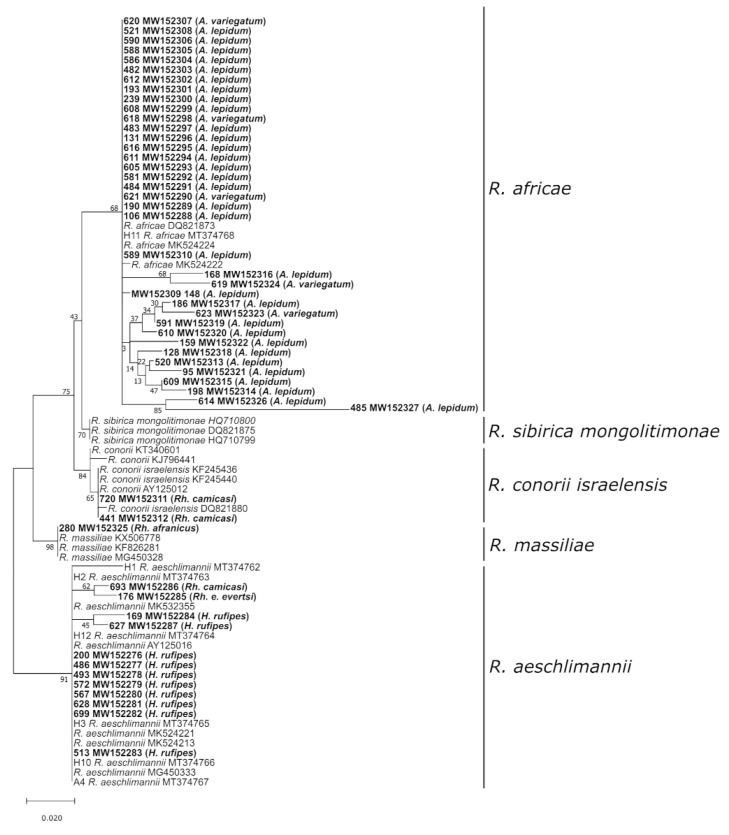
Maximum likelihood phylogenetic analysis of 5S-23S intergenic spacer sequences obtained from *Rickettsia* species. Bolded terminals refer to sequences generated in this study, and sample ID, GenBank accession numbers, and tick host are indicated. Nodal values indicate bootstrap support using 1000 replicates.

In *Amblyomma* spp., the MIR was 12.13% (54/445), and sequencing of the 23S-5S IGS region confirmed *Rickettsia africae* in 37 *Amblyomma* pools (Figure 5). In the remaining 17 samples, the *Rickettsia* DNA content was too low for species identification. The MIR in *Hyalomma* spp. was 4.4% (44/998). Twelve of the 44 samples were successfully sequenced, leading to the identification of *Rickettsia aeschlimannii*. Among the observed *Rhipicephalus* spp., the MIR was 3.93% (38/967). Unfortunately, the majority of *Rickettsia*-positive *Rhipicephalus* samples did not contain enough *Rickettsia* DNA for 23S-5S sequencing. Regardless, *Rickettsia conorii israelensis* was detected in two *Rh. camicasi* pools and *R. aeschlimannii* in one *Rh. evertsi evertsi* pool. The single *Rh. afranicus* specimen was also *Rickettsia*-positive, and subsequent sequencing identified *Rickettsia massiliae*.

#### 3.2.2. Piroplasms

PCR for detection of piroplasms revealed overall MIRs of 0.58% (14/2410) and 0.12% (3/2410) for *Theileria* spp. and *Babesia* spp., respectively (Table 1). Piroplasm species composition among the positive tick pools from North Kordofan and Kassala is shown in Figure 2C.

*Theileria* spp. DNA was detected in *H. anatolicum* (MIR = 2.16%, 4/185), *H. impeltatum* (MIR = 0.67%, 4/600), *Rh. evertsi evertsi* (MIR = 0.88%, 4/454), *Rh. camicasi* (MIR = 0.33%, 1/301), and *A. lepidum* (MIR = 0.26%, 1/387). Five different *Theileria* spp. were identified by 18S rDNA sequencing, as shown in Table 2, including *Theileria ovis, Theileria annulata, Theileria equi, Theileria lestoquardi,* and *Theileria velifera*.

*Babesia* spp. DNA was detected in *H. impeltatum* (MIR = 0.33%, 2/600) and *A. variegatum* (MIR 1.72%, 1/58). The 18S rDNA *Babesia* sequences from *H. impeltatum* showed 98–99% identity to *Babesia pecorum* while the sequences from *A. variegatum* showed 97% identity to *B. caballi* (Table 2).

## 4. Discussion

Ticks and TBDs constitute a global economic and health burden. In countries with a socioeconomic status similar to that of Sudan, a substantial proportion of livestock are owned by subsistence farmers, who are especially vulnerable to the impact of ticks and TBDs [45]. Hassan and Salih [17] stated that the natural population of ticks infesting livestock is changing in Sudan. Therefore, monitoring of the local tick fauna is necessary. In this study, we classified 2410 ticks collected from livestock and other domestic animals in two regions in Sudan into 13 different tick species belonging to the genera *Hyalomma*, *Amblyomma*, and *Rhipicephalus*. Tick screening for *Rickettsia* spp. and piroplasms revealed *Rickettsia* spp., like *R. africae* and *R. aeschlimannii*, as well as several piroplasms of veterinary relevance. Interestingly, we report *Rhipicephalus afranicus*, *Rickettsia conorii israelensis*, *Rickettsia massiliae*, and *Babesia pecorum* for the first time in Sudan. These findings are of high significance for the animal and public health sectors, particularly from a One Health point of view, as rickettsiosis is an important zoonotic disease.

With the exception of *Rh. afranicus*, which can experimentally transmit *Babesia trautmanni* to pigs [29], all of the other detected tick species have formerly been reported in Sudan [15,46,47,48]. Both sampling areas are characterized primarily by Sahelian dry savannah ecosystems; nevertheless, regional differences were noted. Although there was variation in the species composition of the examined host populations, limiting comparability between both regions, the majority of examined animals in both regions were sheep, goats and cattle. Therefore, it was remarkable that the tick fauna in North Kordofan was dominated by *Hyalomma* spp., while *Rhipicephalus* spp. were the most frequent ticks in Kassala. *H. anatolicum*, the main vector of *T. annulata*, which causes bovine tropical theileriosis, has undergone a south- and west-ward spread in Sudan since the 1980s, probably due to animal movement and environmental change [17]. Recently, *H. anatolicum* represented more than 50% of collected ticks in West Darfur, Al-Jazeera, and River Nile states [15]. This indicates that the distribution of *H. anatolicum* has reached the western border of Sudan. In the present study, it was the most frequently observed *Hyalomma* spp. in Kassala, but was also collected in North Kordofan, coinciding with the detection of bovine tropical theileriosis in North Kordofan [49].

In Kassala, *Rh. evertsi evertsi* and *Rh. camicasi* together accounted for more than 50% of collected ticks. Similarly, *Rh. evertsi evertsi* was frequently encountered on cattle in Gezira, central Sudan, and on different domestic animals in West Darfur and River Nile [15]. Both species were also reported by Jongejan, et al. [46] in the Blue and White Nile ecosystems. However, *Rh. camicasi* can be difficult to distinguish morphologically from other ticks of the *Rh. sanguineus* group, which may explain why this species has been less frequently reported in other studies [48,50].

Contrary to the findings of Shuaib, et al. [15], Elghali and Hassan [51] and Ahmed, et al. [50], *A. lepidum* was found in both states, North Kordofan and Kassala, and accounted for approximately 16% of all identified ticks. This tick has historically been abundant in the eastern part of the country, such as Kassala [17]. In recent decades, a westward (towards Kordofan and Darfur regions) spread of *A. lepidum* has been observed [17]. Indeed, the importance of *A. lepidum* lies in the fact that it is the main vector of *Ehrlichia ruminantium*, the causative agent of heartwater, which results in significant morbidities and mortalities in domestic ruminants [52].

One *Rh. afranicus* specimen, collected from a sheep, was identified by sequencing of the 16S rRNA gene. This taxon was historically confounded with *Rhipicephalus turanicus*, however, it was recently described as a distinct species [29]. It has further been confirmed in South Africa [29] and Uganda [53] to date. These *Rh. afranicus* populations may represent two distinct lineages within the species given molecular distances between southern (i.e., South Africa) and northern (i.e., Uganda, Sudan) regions [53]. Interestingly, this specimen carried *R. massiliae* DNA.

The most relevant tick-borne rickettsiae in Africa are *R. africae*, primarily transmitted by *Amblyomma* spp., *R. aeschlimannii*, mainly transmitted by *Hyalomma* spp., and *R. conorii conorii*, which is transmitted by *Rhipicephalus* ticks [54]. In this study, the detected MIR (12.13%) of *Amblyomma* ticks with *Rickettsia* spp. and the confirmation of *R. africae* in the majority of samples denote to a considerable risk of infection of humans with *R. africae*, the causative agent of African tick-bite fever. Similar infection rates of ticks with *R. africae* have been noted in Sudan before [15]. However, high rickettsial infection rates of up to 100% have been described in *Amblyomma* spp. in other regions of Africa, probably due to effective transovarial transmission [54]. Furthermore, MIRS of 4.4% and 3.9% were detected in *Hyalomma* and *Rhipicephalus* ticks, respectively. In previous studies from eastern Africa, these tick genera mostly showed a lower *Rickettsia* prevalence than *Amblyomma* spp., ranging from approximately 10 to 46% in *Hyalomma* spp. [55,56,57] and 0 to 1.1% in *Rhipicephalus* spp. [55,57]. Species identification was only possible in approximately one third of the positive *Hyalomma* pools and the pathogen was confirmed as *R. aeschlimannii*. In the same way, the low rickettsial DNA content did not allow for species identification in most of the *Rhipicephalus* samples. Probably, low rickettsial DNA content is indicative of the fact that the last blood meal of the tick contained rickettsiae, rather than indicating true infection of the tick. Nevertheless, *R. conorii israelensis* was identified in two *Rh. camicasi* pools and *R. aeschlimannii* in one *Rh. evertsi evertsi* pool. *Rickettsia conorii israelensis* is the causative agent of Israeli spotted fever and occurs mainly in the Mediterranean countries [54]. Nevertheless, it has been occasionally detected in Africa, e.g., in Tunisia [58], Nigeria [59] and Kenya [60]. Studies proved that *Rh. sanguineus* s.l. acts as a vector of *R. conorii israelensis,* while the competency of *Rh. camicasi* as a vector is yet to be confirmed [61].

Of note, most of the ticks collected in the present study infest humans only occasionally [62,63]. Nonetheless, this does not rule out the risk of *Rickettsia* spp. transmission to humans. Currently, there are no published data on human *Rickettsia* exposure in Sudan or on the incidence of African tick-bite fever or other rickettsioses. Regarding livestock, high seroprevalences have been observed in sheep (59.3%) and cattle (64.4%) [64]. Considering these high seroprevalence rates and the reported MIRs in this study, investigations into the epidemiology of rickettsiosis in humans are required, concentrating on at-risk populations, especially rural communities with frequent contact with livestock.

Furthermore, relevant tick-borne pathogens for domestic animal health were detected in this study. MIRs were 0.58% for *Theileria* spp. and 0.12% for *Babesia* species. Serological and molecular evidence for circulation of these piroplasms among livestock has been reported in Sudan before [13,65]. Molecular characterization by sequencing of the 18S rDNA revealed that the investigated ticks carried *T. annulata*. In Sudan, bovine tropical theileriosis has been recognized as one of the main limitations that slow the development of the dairy industry [66]. A westward spread of *T. annulata* with its main vector, *H. anatolicum*, has occurred in Sudan, and the pathogen is now also present in North Kordofan, where it was believed to be absent until 2015 [49]. In the present study, we detected *H. anatolicum* in North Kordofan, but all *T. annulata*-positive ticks (one *H. anatolicum* and one *Rh. evertsi evertsi* pool) were from Kassala. Therefore, further studies are needed to assess the risk of *T. annulata* transmission to cattle in North Kordofan.

Overall, the high diversity of pathogenic piroplasms detected in the present study indicates that tick control is relevant for all livestock species in Sudan. Besides *T. annulata*, *T. lestoquardi* that leads to malignant ovine theileriosis, as well as *T. equi* and *B. caballi*, the etiological agents of equine piroplasmosis were also detected, in addition to the apathogenic species *T. ovis* and *T. velifera* [67]. Regarding equine piroplasmosis, *T. equi* was detected in *H. anatolicum* and *B. caballi* in *A. variegatum.* While *H. anatolicum* is a relevant vector for *T. equi*, the detection of *B. caballi* in *A. variegatum* may indicate that this tick had simply ingested infected blood, as *Amblyomma* ticks are not known to act as vectors of *Babesia* spp. [68].

In addition, *B. pecorum* was detected in two *H. impeltatum* pools. This finding suggests that this parasite is globally widespread, since it has been reported in wild animals in South Africa and Spain and in sheep in China [69,70,71]. For transmission of this parasite, *H. anatolicum* showed vector competency in China, whereas in Spain, *H. lusitanicum* was suggested to be the vector of *B. pecorum* [69,71]. It is unlikely that *B. pecorum* is of any relevance for domestic animal health, as experimentally infected non-immunosuppressed sheep and calves did not show any clinical signs [69,71].

## 5. Conclusions

The present study demonstrated a diverse tick fauna on livestock and other domestic animals in Sudan, with *Hyalomma* spp. predominating in North Kordofan and *Rhipicephalus* spp. in Kassala. In addition, the newly described species *Rh. afranicus* was detected. The high *Rickettsia* infection rates indicate a non-negligible risk for humans, especially in pastoral communities and rural areas. The presence of *R. conorii israelensis* in Sudan was documented for the first time. The detection of the highly pathogenic livestock piroplasms (*T. annulata*, *T. lestoquardi*, *T. equi* and *B. caballi*) is an indicator of the need for control programs to reduce the potential economic losses due to ticks and TBDs, as well as for further studies to provide a full picture of their epidemiology.

## Figures and Tables

**Table 1:** Tick species collected from livestock and other domestic animals in Sudan and their pathogen infection rates.

							*Rickettsia* spp.		*Babesia* spp.		*Theileria* spp.	
Tick Species	Total No. of Ticks (% of All Ticks)	Females	Males	Nymphs	No. of Pools	Recorded Host Species	No. of Positive Pools	MIR ^1^ (%)	No. of Positive Pools	MIR ^1^ (%)	No. of Positive Pools	MIR ^1^ (%)
**Genus *Hyalomma***												
*Hyalomma impeltatum*	600 (24.9)	340	260	0	97	camel, cattle, sheep, goat, horse	19	3.17	2	0.33	4	0.67
*Hyalomma anatolicum*	185 (7.68)	71	70	44	89	camel, cattle, sheep, goat, horse, dog	4	2.16	0	0.00	4	2.16
*Hyalomma dromedarii*	136 (5.64)	65	71	0	64	camel, cattle, sheep	6	4.41	0	0.00	0	0.00
*Hyalomma rufipes*	71 (2.95)	16	55	0	32	camel, cattle, sheep, goat	13	18.31	0	0.00	0	0.00
*Hyalomma truncatum*	6 (0.25)	1	5	0	2	cattle	2	33.33	0	0.00	0	0.00
**Genus *Amblyomma***												
*Amblyomma lepidum*	387 (16.06)	99	280	8	115	cattle, sheep	48	12.40	0	0.00	1	0.26
*Amblyomma variegatum*	58 (2.41)	0	58	0	6	cattle	6	10.34	1	1.72	0	0.00
**Genus *Rhipicephalus***												
*Rhipicephalus evertsi evertsi*	454 (18.84)	168	286	0	191	camel, cattle, sheep, goat, horse, dog	10	2.20	0	0.00	4	0.88
*Rhipicephalus camicasi*	301 (12.49)	164	137	0	128	camel, cattle, sheep, goat, dog	14	4.65	0	0.00	1	0.33
*Rhipicephalus decoloratus*	120 (5.00)	120	0	0	23	cattle, sheep, dog	13	10.83	0	0.00	0	0.00
*Rhipicephalus sanguineus* s.l. tropical lineage	89 (3.69)	33	56	0	33	cattle, sheep, dog	0	0.00	0	0.00	0	0.00
*Rhipicephalus microplus*	2 (0.08)	2	0	0	2	cattle	0	0.00	0	0.00	0	0.00
*Rhipicephalus afranicus*	1 (0.04)	1	0	0	1	sheep	1	100.00	0	0.00	0	0.00
**Total**	**2410**	**1080**	**1287**	**52**	**783**		**136**	**5.64**	**3**	**0.12**	**14**	**0.58**

^1^ MIR = minimum infection rate.

**Table 2:** *Babesia* spp. and *Theileria* spp. identified in different tick species collected from livestock and other domestic animals in Sudan from January to August 2017.

Piroplasm	Best Matches in GenBank	Sequence Identity (%), Query Cover (%)	Tick Species	MIR (Positive Pools/ Total Ticks)	State
*Theileria ovis*	MN712508, MN907458, MN704656	99–100, 99–100	*Hyalomma impeltatum*	0.67% (4/600)	North Kordofan
			*Hyalomma anatolicum*	0.54% (1/185)	North Kordofan
			*Rhipicephalus evertsi evertsi*	0.66% (3/454)	North Kordofan, Kassala
			*Rhipicephalus sanguineus*	0.34% (1/293)	North Kordofan
*Theileria annulata*	MN960099, MN907457, MK300062	100, 100	*Hyalomma anatolicum*	1.12% (1/89)	Kassala
			*Rhipicephalus evertsi evertsi*	0.22% (1/454)	Kassala
*Theileria equi*	MN625898, MN611343, MN611344	100, 99	*Hyalomma anatolicum*	0.54% (1/185)	Kassala
*Theileria lestoquardi*	MN704657, KY352037, KP793689	100, 100	*Hyalomma anatolicum*	0.54% (1/185)	Kassala
*Theileria velifera*	LC431550, MH424329, KY450754	100, 100	*Amblyomma lepidum*	0.26% (1/387)	North Kordofan
*Babesia caballi*	MK288110, AB734386, EU642514	97, 98	*Amblyomma variegatum*	1.72% (1/58)	North Kordofan
*Babesia pecorum*	KC249945, KC249944, FJ213577	98–99, 100	*Hyalomma impeltatum*	0.33% (2/600)	North Kordofan
